# If the shoe fits: development of an on-line tool to aid practitioner/patient discussions about ‘healthy footwear’

**DOI:** 10.1186/s13047-016-0149-2

**Published:** 2016-06-07

**Authors:** Lisa Farndon, Victoria Robinson, Emily Nicholls, Wesley Vernon

**Affiliations:** Podiatry Services, Sheffield Teaching Hospitals NHS Foundation Trust, Sheffield, UK; Centre for Women’s Studies, University of York, York, UK; Department of Sociology, University of Portsmouth, Portsmouth, UK

**Keywords:** Footwear, On-line toolkit, Social science methods

## Abstract

**Background:**

A previous study highlighted the importance of footwear to individuals’ sense of their identity, demonstrating that shoes must ‘fit’ someone *socially*, as well as *functionally*. However, unhealthy shoes can have a detrimental effect on both foot health and mobility. This project utilises qualitative social science methods to enable podiatrists to understand the broader contribution of footwear to patients’ sense of themselves and from this an online toolkit was developed to aid footwear education.

**Method:**

Semi-structured interviews were conducted with six podiatrists/shoe-fitters and 13 people with foot pathologies, some of whom also completed shoe diaries. These were supplemented with some follow-up interviews and photographs of participants’ own shoes were taken to allow in-depth discussions.

**Results:**

Four areas related to ‘fit’ were identified; practicalities, personal, purpose and pressures, all of which need to be considered when discussing changes in footwear. These were incorporated into an online toolkit which was further validated by service users and practitioners in a focus group.

**Conclusion:**

This toolkit can support podiatrists in partnership with patients to identify and address possible barriers to changing footwear towards a more suitable shoe. Enabling patients to make healthier shoe choices will help contribute to improvements in their foot health and mobility.

## Background

Foot problems are common in the general population [1] with 61 % of women and 30 % of men report suffering from foot pain [2]. Poorly fitting footwear can be associated with the development of some foot conditions including corns and callus, hallux valgus and lesser toe deformities [3]. Poorly fitting and inappropriate shoes can also lead to falls [4–6]. Information is now available to guide people when choosing suitable footwear as part of the Healthy Footwear Guide [7]. This recommends that a ‘healthy’ shoe should be of adequate width and depth with a toe box and sole that allow for normal foot function. The heel should be stable and approximately 25 mm high and the shoe should keep the foot stable. Despite this marketing figures still suggest a major growth in fashion shoe consumption many of which would not be classed as appropriate according to the guidelines [8]. In this context, podiatrists and patients could currently be seen to be on different ends of a continuum in relation to what is seen as ‘healthy’, desirable footwear from a medical perspective, but which is often perceived as unfashionable and thus unwearable over the long term by patients, despite potential negative implications for their health and well-being.

If it can be shown that the wearing of healthy shoes helps reduce the level or number of painful foot conditions seen, it is possible that the number of people in pain through their foot conditions could be reduced. This would be expected to create improvements in the foot health of the general population and in turn, reduce the burden being placed on NHS podiatry services. This project builds on previous Economic and Social Research Council (ESRC) funded research on the significance of footwear to individuals in terms of identity, transition and memory to examine the motivations, feelings and preferences that affect the footwear choices of patients who are receiving treatment and support from Podiatry Services [9]. The purpose of this study was to use qualitative methods (via interviews and a focus group) to design a practical on-line toolkit to empower foot health practitioners to encourage healthier shoe choices in the people they treat. If more successful strategies can be used to persuade people to change their footwear in turn foot health and mobility could be improved.

## Methods

A variety of recruitment mechanisms were used in order to reach out to both professionals and patients. Patient recruitment was targeted primarily in Yorkshire and the North East. Flyers and posters were made available in clinics, and eligible potential participants were also identified by practitioners and invited to consider participation. Information about the study was circulated at local diabetes support groups and social media was used extensively to promote the study, specifically through the development of a widely-circulated Facebook page. Practitioners were recruited through targeted advertising in Podiatry Now magazine and through circulation of emails to members of The College of Podiatry and other relevant mailing lists.

Semi-structured interviews were conducted with six podiatrists/shoe-fitters and 13 people with a range of foot pathologies including corns, callus and foot deformities; some of whom also completed shoe diaries. Participants were asked to complete a shoes diary for 1 month, detailing each day what shoes they wore, what the occasion was and how they were travelling (if applicable) e.g. cycling, walking, driving, using public transport. The podiatrists and shoe-fitters were all experienced in footwear education and had all been practising over 10 years. The initial interviews were supplemented with some follow-up interviews and photographs of participants’ own shoes were taken to allow and guide more in-depth discussions. The interviews were carried out to answer the following questions:

1. What factors shape and motivate the footwear choices and preferences of patients receiving treatment and support from Podiatry Services?
2. Where does conflict arise in negotiations between patients and podiatrists around healthy footwear choices?
3. How can these tensions be addressed through providing training to facilitate dialogue between patients and podiatrists?

All interviews were recorded, transcribed and transcripts and shoe diaries manually coded into themes and sub-themes. Each theme was analysed and summarised. Mapping out all the different themes present across the multiple means of data collection provided a template from which to build and develop the toolkit to ensure it was well-grounded in the data. Focus group and field trial data was used to further refine the on-line toolkit. The toolkit consists of details and findings from the study including a visual tool of recommendations for practice. It also includes information for patients and links to the Healthy Footwear Guide, The Society of Shoe Fitters and Sheffield Foot and Ankle Pain website.

Once the toolkit was formulated it was presented in a focus group of service users and practitioners to provide feedback and allow for final modifications.

These methods were deemed a highly appropriate means through which to engage both health professionals and the people they treat. It is hoped that the links between shoes, wellbeing and identity can be explored as rich data would be expected to be generated. Also the potential to explore all perspectives in more depth would be possible using qualitative research methods.

## Results

**Fig. 1:**
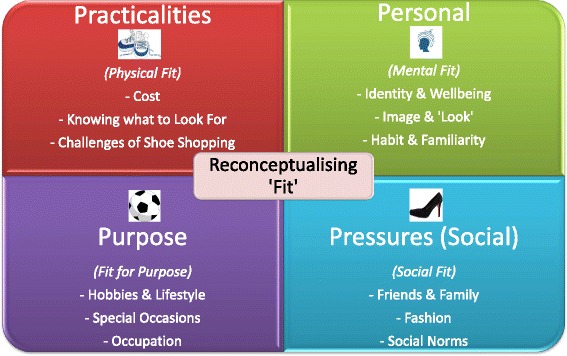
Visual Representation of the toolkit

Initial themes and sub-themes are illustrated in Table 1. Further refinement of these themes led to the development of a visual tool to aid practitioners when discussing footwear changes with patients (Fig. 1).

**Table 1:** Themes and sub-themes generated from the interviews and focus group

Theme	Sub-theme
Comfort/Pain management	Factor on which most shoe choices are based
	Physical ‘fit’
	Habit/familiarity
	Wearing similar or same shoe almost every day
	Enjoyment gone from shoes
Fashion	At odds with comfort
	Used to be important
Cost	High cost of shoes prohibitive
	Begrudges cost
	People with disabilities are ‘exploited’/discriminated against in terms of footwear choices and costs
	What they pay
	Orthotics/specialist shoes
Identity	Sense of shoes not matching self/lifestyle
	Mental ‘fit’
	Medicalisation—don’t want to be seen as someone with a disability etc.
	Pride, wellbeing and self-esteem
	Unhappy with shoes/wishes could wear different shoes
	Ageing
	Gender
Image	Creating a ‘look’ or a brand/image
	Different outfits
	Hates feet and having them on display
	Minimise visibility of shoes
	Colour
Occasions	‘Appropriateness’
	Formal shoes
	Season/weather
	Work
	Socialising/leisure
	Getting out and about
Shoe-shopping	Hard to find wide-fit shoes/shoes that fit
	Shoe shopping has to be planned now
	Limited access to shops
	Embarrassing/hates it
	Prefer to shop online/catalogues or try shoes on at home
	Staff lack knowledge to help
Peer pressure	
What makes a healthy shoe?	
Support from Podiatrists	Good support and advice
	Didn’t realise shoe advice part of pod role/never received it
	Changing role of podiatrists/professionalisation
	Training
What would help?/recommendations for toolkit and for changing footwear	Offer examples of specific brands
	Recognise patient as individual with individual needs
	Get to know the patient and build rapport/build concordance
	Understand mental wellbeing side of shoes/people’s feelings and emotions
	LOCAL context—shops in area
	Explain things more clearly
	Show images of shoes
	Shoe-fitting service
	Shoe-fitting/foot-measuring in shops
	Private area in shops
	Empowerment/choice
	Knowing what pods do more
	Internet/peer support
	Not treating patients who won’t make the changes
	Gradual/step changes
	Balance in managing situation
	‘Car to bar’—changing shoe choice some of the time
	Role of prevention
	People don’t make the connection between foot conditions and shoes
	Change people’s perceptions of healthy shoes?
	Look at the bigger picture

### Conflict

**Fig. 2:**
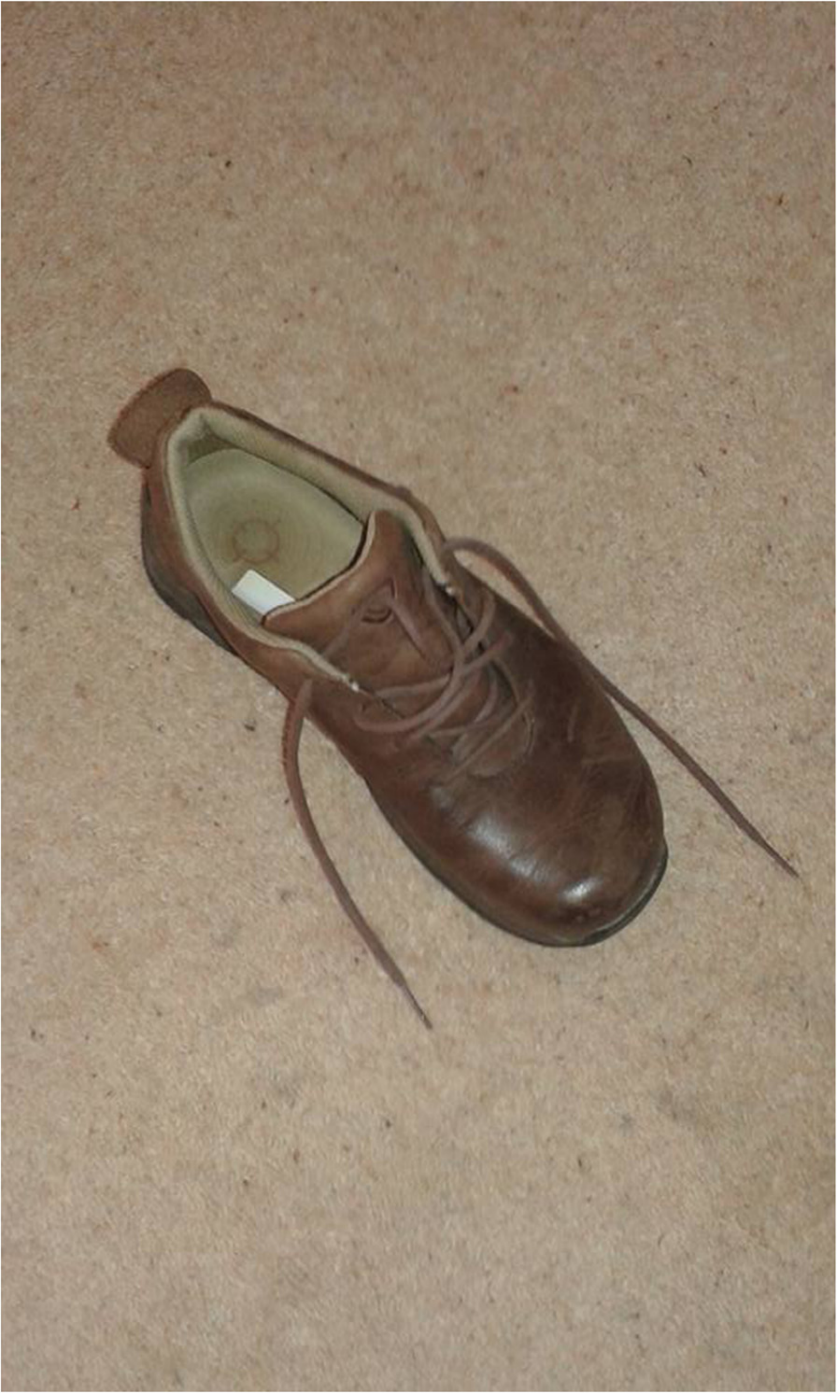
Nigel’s shoe

There can be conflict in what podiatrists and the people that they support and treat look for in a shoe, with many service users raising the importance of the visual appearance of footwear and also the links between footwear, occasion and identity. These themes arose consistently across patient interviews, and there was often considerable overlap between them. For example, Nigel, a recently-diagnosed diabetic in his 40s, talked extensively about the difficulties he experienced in finding suitable footwear, and the importance of the ‘right’ shoes to his wellbeing, self-esteem and pride, as well as his sense of masculinity. The unfashionable ‘fuddy duddy’ shoes he was required to wear in order to accommodate his ulcers and foot pain inhibited his perceived ability to present himself as ‘smart’ and ‘trendy’ and resulted in him feeling socially isolated from particular occasions and describing a part of his identity as being ‘missing’ (Fig. 2). This tension between ‘comfort’ and ‘fashion’ in shaping shoe choices was evident across the majority of patient interviews, as were discussions around the particular ‘barriers’ to selecting appropriate footwear that were frequently encountered.

These barriers were grouped into four distinct themes around the concept of ‘fit’:

#### Practicalities (physical fit)

When purchasing appropriate footwear a number of practical considerations were raised by the respondents including cost. Many assumed that healthy footwear is expensive, or contrary to this, the more expensive the shoe the better it must be. Balancing comfort and fit with the cost of the shoe was a common issue for participants, as illustrated by Frank and Charlie, both diabetics in their fifties who were interviewed together and expressed concern about what they saw as the prohibitive costs of appropriate footwear:

*Interviewer: Would you say that that’s the main priority then… comfort?*

*Charlie: Yes.*

*Frank: Comfort, yeah, definitely. And price as well because I’m on benefits. Because some of the wide-fitting shoes are ridiculous prices.*

*Charlie: Yeah, I mean I’m the same, it’s got to be the right price range to fit my pocket and my feet at the same time, do you know?*

There were also difficulties in knowing what to look for in a good shoe, which stockists to purchase them from and actually getting to certain shops for some with mobility problems. Participants felt pictures of ‘healthy’ footwear and lists of local stockists could help with this. Difficulties were also discussed when actually trying to buy shoes in terms of choice and styles which may be limited if feet have specific deformities. The problems of purchasing footwear online were also highlighted, as it is difficult to ascertain fit and comfort until shoes have been tried on.

#### Personal (mental fit)

Footwear was associated with an individual’s identity and personality. Shoes are related to image and should ‘fit’ someone mentally as well as physically or they will not be worn. Those who had to wear shoes that they felt were unattractive said that this could affect their self-esteem, self-image and pride. For example, Jo – who was in her thirties - had been advised to wear a specific brand of trainers by her podiatrist, but felt this ‘clashed’ with her desire to present herself as an ‘alternative’ person and did not fit with the image and identity she wished to portray through her dress and appearance:

*…people who wear these shoes [trainers] conform to a lifestyle that I do not conform to, so I feel a bit like a pretender wearing them as well. I feel that they hint to a lifestyle that I do not want to be associated with’*

Many respondents commented that changing shoe buying and wearing habits were difficult, even if advised by a podiatrist. The positive benefits of changing footwear on foot health should be encouraged rather than emphasising the negative serious problems that may occur.

#### Purpose (lifestyle fit)

It is important that footwear is appropriate in terms of lifestyle, hobbies and occupational needs. When recommending shoes, practitioners should take into consideration the social norms and expectations around footwear and the impact these might have on individuals’ preferences and values. Some participants commented that they were in jobs that required a certain ‘type’ of shoe which would be difficult to change. For example, Tricia, a retired solicitor with foot pain and plantar fasciitis, talked extensively about the difficulty of managing her footwear choices throughout her career. Special occasions and events could also be particularly difficult times to manage shoe choices, as illustrated by Elizabeth, a great grandmother in her eighties who was very resistant to attempts by podiatrists to encourage her to wear more supportive shoes:

*Interviewer: How do you think you’d feel if you were wearing something like that [Velcro-fastening shoe recommended by Podiatrist] to a formal occasion?*

*Elizabeth: I wouldn’t. There is no way. I’d sorted all my shoes out and I’d thrown them away and then I realised that my great-granddaughters were being christened and I thought ‘God’! So I had to go out and buy a pair of court shoes because, I said to my daughter, ‘there’s no way I can go to the christening, you know, with my feet like this… so I’ve been out and I’ve bought a pair of shoes’*

#### Pressures (social fit)

A number of pressures could affect footwear choice and restrict change; these include fashion and social norms. Unsurprisingly, fashion was important—at least to an extent—for the majority of participants regardless of gender, and many felt there was always a significant disconnect between ‘fashion’ and ‘comfort’. Peer pressure also played a role in shaping shoe choices, including occasional judgement or negative reactions when participants attempted to change their shoe choices, as illustrated by Ron, a man with diabetes in his seventies with foot swelling and discomfort:

*I had some a while ago with the Velcro fastening and people laughed at them. My son in particular said ‘you look ridiculous wearing those shoes'*

It is clearly important for practitioners to recognise this and where required, to work to bring the wider family ‘on board’ with any proposed footwear changes.

These themes formed the basis of the toolkit to help reconceptualise ‘fit’ in ways that reflect the wider understanding of the term apparent amongst the participants with foot pathologies (Fig. 1).

## Discussion

The links between foot pathologies and inappropriate shoes is well established [3] despite this it is still often difficult for people to obtain footwear that fits properly [10]. This is exacerbated in those with specific foot deformities such as rheumatoid arthritis [11] and diabetes where correct footwear is often key to reducing the development or recurrence of foot deformities and pathologies. From this investigation in the context of footwear, *fit* is more than just physical. Whilst a ‘healthy’ shoe may be one that is perceived to fit well physically, results from this investigation indicate the importance of re-thinking what we mean by ‘fit’ and imagining it in a wider sense, in terms of practicalities, personal, purpose and pressures. Goodacre et al. [12] found that when compromises have to be made in footwear choice to accommodate specific foot conditions in rheumatoid arthritis; identity and social status can be affected. This can lead to poor compliance when wearing specialist orthopaedic footwear due to its ‘look’ [13]. These themes were also found in this study where shoes should be able to ‘fit’ the person both mentally and socially if they are going to be worn.

It is hoped that this toolkit can act as a prompt or trigger for discussion in direct consultation with patients, for example in combination with techniques such as motivational interviewing (MI). MI techniques have been shown to effect lifestyle behaviour changes such as weight management and alcohol consumption [14]. Using MI could help patients to identify how far the different types of fit and related barriers impact upon their own shoe choices working through the visual representation of the tool. This could help podiatrists to understand the values and motivations of their patient’s and to open up further dialogue and discussion around the next steps forward and how to make changes.

In order to further help with footwear change, practitioners could provide examples of different types of shoes either in picture format or using actual shoes to help to challenge this pre-conception that fashion and comfort are at different ends of the scale. Footwear recommendations should be tailored to take into account individual’s lifestyles but with the emphasis on how ‘healthy’ shoes could improve mobility and reduce pain.

The next stage of this work will be to ‘test’ the effectiveness of the toolkit in a large group of patients to investigate if it can help guide and change footwear choices in a podiatry practice context.

## Conclusions

This research suggests that there are some areas of common ground between practitioners and patients on which to build. To maximise the chances of individuals making footwear changes, foot health professionals must work to address these four barriers that service users may encounter. Thinking more holistically about ‘fit’ allows foot health professionals to take into account individuals’ preferences and values, psychological and emotional wellbeing and the ways social pressures impact on footwear decisions. Consideration of fit in this wider sense will improve interaction between practitioner and service user and increase the likelihood that positive, long-term and sustainable footwear changes are made to make ‘every contact count’.

Both the visual tool and recommendations are available to download and print on the study website (www.sheffield.ac.uk/podiatrytoolkit).

## Abbreviations

Not applicable
